# Distribution and Seasonal Activity of Phlebotominae Sand Flies in Yazd and Its Outskirts, Center of Iran

**DOI:** 10.1155/2017/1486845

**Published:** 2017-12-27

**Authors:** Motahareh Mirhoseini, Aref Salehzadeh, Sara Ramazan Jamaat, Amir Hosein Zahirnia, Najmeh Rahmanzadeh

**Affiliations:** ^1^Department of Medical Entomology and Vector Control, School of Medicine, Hamadan University of Medical Sciences, Hamadan, Iran; ^2^Department of Biostatistics, School of Public Health, Hamadan University of Medical Sciences, Hamadan, Iran; ^3^Yazd Health Center, Yazd, Iran

## Abstract

**Background:**

Phlebotominae sand flies are the main vectors of leishmaniasis and some other diseases.

**Materials and Methods:**

Using sticky traps, sand flies were collected fortnightly from outdoors and indoors areas of selected sites.

**Results:**

A total of 2032 specimens (498 in the city and 1534 in the outskirts of Yazd) belonging to 11 species were collected. The activity of sand flies started in early-April and ended in mid-November. There were two peaks of activity in the end of April and mid-September.* Phlebotomus sergenti* and* P. papatasi* were the most abundant species in the city and outskirts of Yazd city, respectively. Other species were* P. salehi*,* P. ansarii*,* P. kazerouni*,* P. caucasicus*,* P. andrejevi*,* P. alexandri*,* P. mongolensis*,* Sergentomyia sintoni,* and* S. palestinensis*.

**Conclusion:**

In comparison to some other parts of Iran, the extended period between two peaks suggests that the larvae to adult development of sand fly were delayed by the higher temperature of the summer months in Yazd province.

## 1. Introduction

Phlebotominae sand flies are placed in the family Psychodidae. There are more than 900 species and subspecies of sand flies worldwide [1] and the presence of at least six genera,* Phlebotomus* (Rondani and Berté, 1840),* Sergentomyia* (França and Parrot, 1920), and* Chinius* (Leng 1987) in the old world and* Brumptomyia* (França and Parrot, 1921),* Lutzomyia* (França, 1924), and* Warileya* (Hertig, 1948) in the new world has been accepted by most specialists such as Bates et al. [1] and Akhoundi et al. [2]. Leishmaniasis refers to a set of zoonotic diseases usually transmitted by blood-feeding female sand flies infected with flagellate protozoan of the genus* Leishmania *(Ross, 1903). The disease covers a wide range of clinical manifestations, from self-healing lesion that is known as localized cutaneous leishmaniasis (CL) to a severe systemic form or visceral leishmaniasis (VL) in which the parasites migrate to the vital organs and if not treated in time can lead to death. Although cutaneous leishmaniasis is not a fatal disease, but due to the long term treatment process and otherwise involvement of patients with chronic wounds and scars, it has always been of particular concern for people in endemic areas. Many studies have been conducted in the field of leishmaniasis and there is reduction in the number of reported cutaneous cases of leishmaniasis in Iran [3, 4], but even with the great efforts of health authorities over the last four decades, there is still a considerable number of foci of disease distributed throughout the country [4]. In addition to different types of leishmaniasis, sand flies may carry a number of other viral diseases such as papatasi fever, summer meningitis, or Toscana virus as well as Carrion's disease that is caused by bacteria [5, 6]. Similar to most other studies on arthropod borne diseases on the fauna of vectors, the distribution and other characteristics of diseases have great importance in planning any program to reduce or even stop transmission. The present study was designed to determine some factors which affect distribution and population size of Phlebotominae sand flies in Yazd city and the suburbs of Yazd.

## 2. Materials and Methods

**Figure 1:**
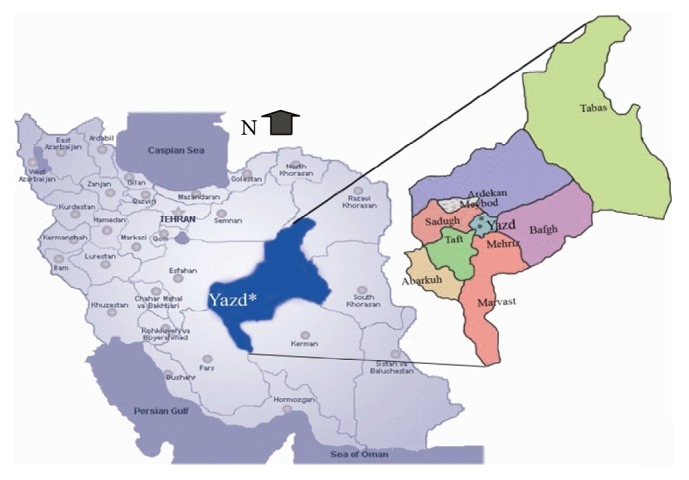
Map of study area.

**Figure 2:**
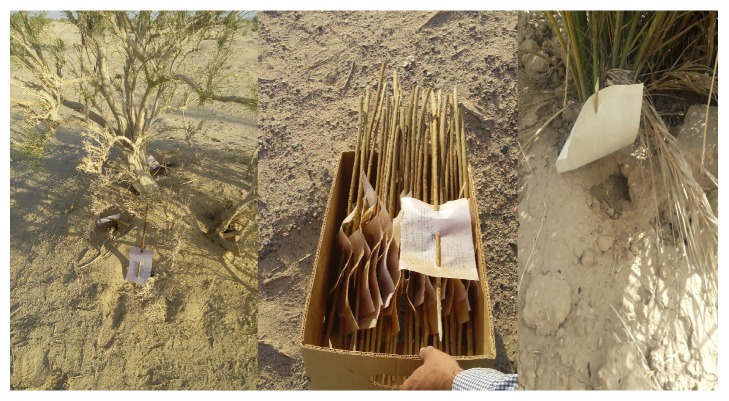
A sampling site in the outskirts of Yazd (the burrows of gerbils can be seen in the picture).

This is a cross-sectional study on the distribution and species diversity of sand flies of Yazd and its outskirts, conducted in 2015. Five collection spots were utilized and in each spot two stations in different regions of Yazd (North, South, East, West, and Center) were selected. The traps were placed throughout the season of activity of sand flies from the second half of March to mid-November, once every 15 days (a total of 17 times) and 60 traps for each time (30 indoor traps and 30 outdoor traps). In order to compare species diversity in the city and suburb of Yazd, including villages Sadr Abad, Haji Abad, Bafgh, Abarkuh, and Nosratabad, trapping was conducted in these areas as well. The geographical location of the collection sites was recorded using Global Positioning System (GPS) Garmin Oregon 600. Also the map of study area has been shown in Figure 1. Sticky traps were put in collection sites including animal burrows, near mud walls, basements of old buildings, and other places likely to harbor sand flies before sunset and collected before sunrise (Figure 2). Then the traps were transferred to the laboratory to determine the species. Sand flies were removed from the trap with the help of a micro dissecting needle in the laboratory and placed in acetone for degreasing. Thereafter, the samples were mounted on a glass slide using Puri glue and a cover slide. After 48 hours, using taxonomic keys and comparison with species of the standard collection the samples were identified to the extent possible according to the keys described by Nadim and Javadian [7], Lewis [8], Rassi and Hanafi-Bojed [9], and Artemiev and Neronov, [10]. In order to assess the effect of status of buildings and the plant coverage of the study areas on the density of sand flies, the characteristics and age of building and also the status of plant coverage were recorded (areas with few scattered plants grouped in uncovered area).

## 3. **Results**

During the period of study, a total of 2031 Phlebotominae sand flies were collected. There were 10 species of Phlebotominae identified in two* Phlebotomus* and* Sergentomyia* genera. Only 498 specimens were from Yazd city and 1533 of them were from the outskirts of Yazd. Although* Phlebotomus papatasi* was the most abundant species, this species was collected only in outskirts of Yazd. Also,* P. sergenti*,* P. salehi*,* P. mongolensis*,* P. alexandri,* and* S. palestinensis* were collected only in outskirts of Yazd (Table 1).

**Figure 3:**
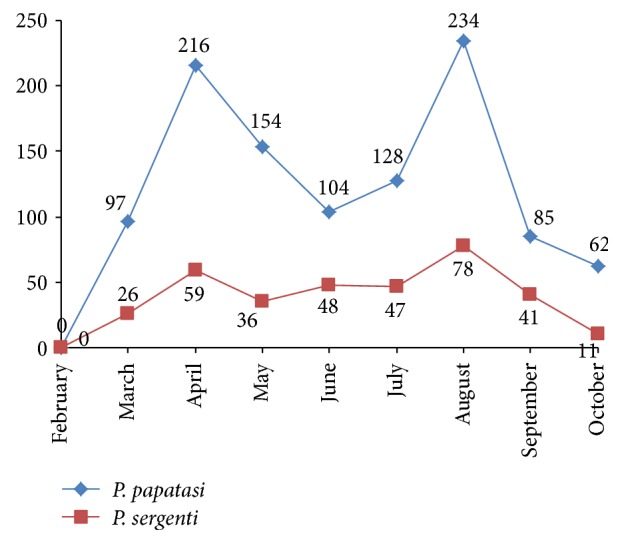
Sand flies seasonal activity in Yazd and its outskirts, 2015.

The activity of sand flies started in early-April and ended in mid-November. There were two peaks of activity in the end of April and mid-September (Figure 3).* Phlebotomus papatasi*,* P. sergenti,* and* S. sintoni* were collected in all active months. They were present at both the beginning and ending of the active season.* Phlebotomus caucasicus* was the only species with male/female sex ratio less than one (Table 1).

The comparison of specimens captured in areas dominated either with old or new buildings of Yazd showed that sand flies were more abundant in areas dominated with old and mud buildings (Table 2).

Also, a comparison of Phlebotominae density in areas covered with plants and uncovered areas or areas with few scattered plants showed that sand flies in plant covered areas were more abundant (Table 3).

Although* P. sergenti* had the highest population in Yazd, the overall dominant species in the study areas were* P. papatasi* and* S. sintoni* with 53% and 25% of all collected specimens, respectively. Also* P. andrejevi, P. kazerouni, *and* P. alexandri* had the lowest frequency each with only one specimen (Table 1).

## 4. Discussion

The result of this study showed that the activity of sand flies in Yazd starts in early-April and ends in mid-November and there were two peaks of activity, one at the end of April and the other in late September. In comparison to some other studies in Iran such as the studies of Salehzadeh et al. [11] and Azizi et al. [12] which were carried out in regions with more temperate climate, one of the most important differences is the long distance between the first and second peaks of Phlebotominae activity in Yazd. Such distance between two activity peaks of sand flies has also been reported in Sistan-Baluchistan Province, Southeast Iran [13]. In this case, it seems that the larvae to adult development of Phlebotominae were delayed by the hot and dry summers of Yazd and Sistan-Baluchistan provinces.

Regarding the length of the active season, the comparison of Yazd with cities such as Jask in Hormozgan province in the south of Iran [14] that has a milder and more humid weather shows that the active season of sand flies in Jask is longer than the active season of sand flies in Yazd since the activities of sand flies in Jask start from late March and continue to late November. This means that a longer warm season and higher humidity may cause longer active season. On the other hand, comparing Yazd with cities such as Ali Abad Katul, Golestan province, North of Iran [15], with relatively colder climate shows that the activity of sand flies starts in early June and continues until mid-October and so is shorter than Yazd which reiterates the effect of environmental condition on the duration of the active season of sand flies.

In the present study, 10 species were identified (Table 2).* Phlebotomus sergenti* and* Phlebotomus papatasi* are the main vectors of anthroponotic cutaneous leishmaniasis and zoonotic cutaneous leishmaniasis, respectively, in many parts of the old world. Natural infection to* Leishmania* parasites also has been reported in* P. caucasicus*,* P. salehi*,* P. mongolensis*,* P. ansarii*,* P. salehi*,* P. alexandri,* and* P. andrejevi* [16], which shows their importance in the maintenance of diseases.

In comparing the species diversity of sand flies in Yazd city and the outskirts of Yazd, one of the interesting points here is that although* P. papatasi* is a predomestic species and can be seen in almost all Phlebotominae infested domestic areas, despite the numerous trapping in the activity season, 17 sets of collection did not show any specimen of this species in Yazd city. In this case the absence of* P. papatasi* in Yazd may suggest a higher relative sensitivity of this species to pesticides because of pesticide application in Yazd (while interacting with health authorities of Yazd, they revealed that pesticides are currently being used to combat cutaneous leishmaniasis). This also can explain more diverse species and the higher number of sand flies captured in the outskirts of Yazd.

On the other hand* P. sergenti* was not captured in the outskirts of Yazd which is dominated with rodent burrows. The abundance of* P. papatasi* and absence of* P. sergenti* in the outskirts of Yazd may also indicate the lower tendency of* P. sergenti* to rodent burrows or* Haloxylon* (a plant that has been cultivated for reconditioning of the desert). Similar to other studies [6, 11] which used the sticky traps to collect Phlebotominae sand flies male/female sex ratio for most species was more than one, except for* P. caucasicus *which was less than one. Such differences were also reported in the case of* P. duboscqi* in Northern Ethiopia [17].

The similarity of seasonal activity of prevalent species in its Yazd and outskirts (*P. papatasi* and* P. sergenti*) demonstrated in Figure 3 suggests that weather is probably the most important factor in determining the seasonal activity of these flies, because weather is the only factor that is almost identical in Yazd and the outskirts of Yazd.

Tables 3 and 2 show the effect of plant coverage and status of buildings on density of sand flies. These results show that plant coverage especially in a region like Yazd with annual fall of 60 mm is very essential to the survival of these insects. The importance of plant coverage and the presence of mud walls are also suggested by other authors as well [18].

Another interesting point is the difference in type of leishmaniasis in Yazd and the outskirts of Yazd, because Yazd is foci of anthroponotic cutaneous leishmaniasis [19] and* P. sergenti,* the vector of urban leishmaniasis, was the most prevalent species in Yazd, whereas zoonotic cutaneous leishmaniasis is mostly reported from the outskirts of Yazd [20] which had the highest population of* P. papatasi,* the most important vector of rural cutaneous leishmaniasis. In this case, the presence of plants such as Haloxylon and gerbil colonies are two important ecological factors in the zoonotic foci of cutaneous leishmaniasis [21].

The results presented here clearly show the relationship between the distribution of different types of cutaneous leishmaniasis and fauna of Phlebotominae.

With regard to the presence of two peaks of activity of sand flies in Yazd, it is more likely for humans to be bitten in these two periods; therefore, preventive measures must be taken shortly before the aforementioned dates. Also due to the activity of some important suspected vectors in this area, molecular investigations to elucidate the status of infection in sand flies and the reservoir host are recommended.

## Figures and Tables

**Table 1:** Distribution of *Phlebotomus* and *Sergentomyia* species in Yazd city and the outskirts of Yazd, 2015.

Species	Yazd		Outskirts of Yazd		Male/female sex ratio	Total
	Female	Male	Female	Male		
*P. sergenti*	95	256	0	0	2.694	351
*P. caucasicus*	11	3	22	4	0.181	40
*P. andrejevi*	0	1	0	0	—	1
*P. ansarii*	0	1	0	15	—	16
*P. kazeroni*	0	0	0	1	—	1
*P. papatasi*	0	0	274	806	2.941	1080
*P. alexandri*	0	0	2	4	2	6
*P. salehi*	0	0	2	11	5.5	13
*P. mongolensis*	0	0	0	16	—	16
*S. sintoni*	24	107	147	229	1.964	507

**Table 2:** The effect of buildings status on density of sand flies captured in Yazd city, 2015.

Status of building	*P. andrejevi*	*P. ansarii*	*P. caucasicus*	*S. sintoni*	*P. sergenti*
Old	(1)	(1)	84.6% (110)	92.3% (12)	70.7% (176)
New	0	0	15.4% (20)	7.7% (1)	29.3% (73)

**Table 3:** The effect of plant coverage on density of sand flies captured in Yazd city, 2015.

Plant coverage	*P. andrejevi*	*P. ansarii*	*P. caucasicus*	*S. sintoni*	*P. sergenti*
Covered areas	%100	%100	%76.9	%54.6	%68.4
	(1)	(1)	(10)	(71)	(238)
Uncovered areas	0	0	%23.1	%45.4	%31.6
			(3)	(59)	(110)

## References

[1] Bates P. A., Depaquit J., Galati E. A. B. (2015). Recent advances in phlebotomine sand fly research related to leishmaniasis control. *Parasites & Vectors*.

[2] Akhoundi M., Kuhls K., Cannet A. (2016). A Historical Overview of the Classification, Evolution, and Dispersion of Leishmania Parasites and Sandflies. *PLOS Neglected Tropical Diseases*.

[3] Abedi-Astaneh F., Hajjaran H., Yagoo bi-Ershadi M. R. (2016). Risk mapping and situational analysis of cutaneous leishmaniasis in an endemic area of central iran: a gis-based survey. *PLoS ONE*.

[4] Norouzinezhad F., Ghaffari F., Norouzinejad A., Kaveh F., Gouya M. M. (2016). Cutaneous leishmaniasis in Iran: Results from an epidemiological study in urban and rural provinces. *Asian Pacific Journal of Tropical Biomedicine*.

[5] Es-Sette N., Ajaoud M., Anga L., Mellouki F., Lemrani M. (2015). Toscana virus isolated from sandflies, Morocco. *Parasites & Vectors*.

[6] Rafatbakhsh-Iran S., Salehzadeh A., Nazari M. (2016). Ecological Aspects of the Predominant Species of Phlebotominae Sand Flies (Diptera: Psychodidae) in Hamadan, Iran. *Zahedan Journal of Research in Medical Sciences*.

[7] Nadim A., Javadian E. (1976). Key for species identification of sandflies (Phlebotominae; Diptera) of Iran. *Iranian Journal of Public Health*.

[8] Lewis D. J. (1982). A taxonomic review of the genus Phlebotomus (Diptera: Psychodidae). *Bulletin of the British Museum (Natural History)*.

[9] Rassi Y., Hanafi-Bojed A. (2006). *Sandflies, Leishmaniasis vectors*.

[10] Artemiev M. M., Neronov V. (1984). Distribution and ecology of sandflies of the world (genus Phlebotomus). *Institute of Evolution, Morphology and Animal Ecology*.

[11] Salehzadeh A., Iran S. R., Latifi M., Mirhoseini M. (2014). Diversity and incrimination of sandflies (Psychodidae: Phlebotominae) captured in city and suburbs of Hamadan, Hamadan province, west of Iran. *Asian Pacific Journal of Tropical Medicine*.

[12] Azizi K., Parvinjahromi H., Moemenbellah-Fard M. D., Sarkari B., Fakoorziba M. R. (2016). Faunal distribution and seasonal bio-ecology of naturally infected sand flies in a new endemic zoonotic cutaneous Leishmaniasis focus of southern Iran. *Journal of Arthropod-Borne Diseases*.

[13] Kassiri H., Javadian E., Sharififard M. (2013). Monthly activity of phlebotominae sand flies in sistan-baluchistan province, Southeast Iran. *Journal of Insect Science*.

[14] Azizi K., Fekri S. (2011). Fauna and bioecology of sand flies in Jask country, the endemic focus of cutaneous leishmaniasis in Hormozgan, Iran. *Bimonthly Journal of Hormozgan University of Medical Sciences*.

[15] Bagheri A., Sofizadeh A., Ghezel A., Ghanbari M., Fadaei E., Cherabin M. (2011). Ecological characteristics of sand flies in Golestan province. *Journal of Gorgan University of Medical Sciences*.

[16] Yaghoobi-Ershadi M. (2012). Phlebotomine sand flies (Diptera: Psychodidae) in Iran and their role on *Leishmania* transmission. *Journal of Arthropod-Borne Diseases*.

[17] Gebresilassie A., Kirstein O. D., Yared S. (2015). Species composition of phlebotomine sand flies and bionomics of *Phlebotomus orientalis* (Diptera: Psychodidae) in an endemic focus of visceral leishmaniasis in Tahtay Adiyabo district, Northern Ethiopia. *Parasites & Vectors*.

[18] Bern C., Courtenay O., Alvar J. (2010). Of cattle, sand flies and men: a systematic review of risk factor analyses for South Asian visceral leishmaniasis and implications for elimination. *PLOS Neglected Tropical Diseases*.

[19] Yaghoobi-Ershadi M. R., Hanafi-Bojd A. A., Javadian E., Jafari R., Zahraei-Ramazani A. R., Mohebali M. (2002). A new focus of cutaneous leishmaniasis caused by *Leishmania tropica*. *Saudi Medical Journal*.

[20] Yaghoobi-Ershadi M. R., Jafari R., Hanafi-Bojd A. A. (2004). A new epidemic focus of zoonotic cutaneous leishmaniasis in central Iran. *Annals of Saudi Medicine*.

[21] Ghaffari D., Hakimi Parizi M., Yaghoobi Ershadi M. R., Sharifi I., Akhavan A. A. (2014). A survey of reservoir hosts in two foci of cutaneous leishmaniasis in Kerman province, southeast of Iran. *Journal of Parasitic Diseases*.

